# SGLT2 Inhibitors in Cancer Patients: A Comprehensive Review of Clinical, Biochemical, and Therapeutic Implications in Cardio-Oncology

**DOI:** 10.3390/ijms26104780

**Published:** 2025-05-16

**Authors:** Alessandra Greco, Maria Laura Canale, Vincenzo Quagliariello, Stefano Oliva, Andrea Tedeschi, Alessandro Inno, Marzia De Biasio, Irma Bisceglia, Luigi Tarantini, Nicola Maurea, Alessandro Navazio, Marco Corda, Attilio Iacovoni, Furio Colivicchi, Massimo Grimaldi, Fabrizio Oliva

**Affiliations:** 1 Cardiology Division, Fondazione IRCCS Policlinico San Matteo, 27100 Pavia, Italy; a.greco@smatteo.pv.it; 2 Cardiology, Versilia Hospital, Azienda USL Toscana Nord-Ovest, 55041 Lido di Camaiore, Italy; marialaura.canale@uslnordovest.toscana.it; 3 Cardiology Division, Istituto Nazionale Tumori, IRCCS Fondazione G. Pascale, 80145 Naples, Italy; 4 UOSD Cardiologia di interesse oncologico, IRCCS Istituto Tumori “Giovanni Paolo II”, 70124 Bari, Italy; s.oliva@oncologico.bari.it; 5 Cardiology, “Guglielmo da Saliceto” Hospital, 29121 Piacenza, Italy; andrea.tedeschimd@gmail.com; 6 Medical Oncology, IRCCS Ospedale Sacro Cuore Don Calabria, Negrar di Valpolicella, 37024 Verona, Italy; alessandro.inno@sacrocuore.it; 7 Cardiology, Azienda Sanitaria Universitaria Friuli Centrale, 33100 Udine, Italy; marzia.debiasio@asufc.sanita.fvg.it; 8 Servizi Cardiologici Integrati, Dipartimento di Scienze Cardio-Toraco-Vascolari, Azienda Ospedaliera San Camillo Forlanini, 00148 Rome, Italy; irmabisceglia@gmail.com; 9 S.O.C. Cardiologia Ospedaliera, Presidio Ospedaliero Arcispedale Santa Maria Nuova, Azienda USL di Reggio Emilia-IRCCS, 42100 Reggio Emilia, Italy; luigi.tarantini@gmail.com (L.T.); alessandro.navazio@ausl.re.it (A.N.); 10 S.C. Cardiologia, Azienda di Rilievo Nazionale e Alta Specializzazione “G. Brotzu”, 09047 Cagliari, Italy; marcocorda2@aob.it; 11 SSD Chirurgia dei Trapianti e del Trattamento Chirurgico dello Scompenso, Dipartimento Cardiovascolare, ASST Papa Giovanni XXIII, 24127 Bergamo, Italy; aiacovoni@asst-pg23.it; 12 UOC Cardiologia Clinica e Riabilitativa, Presidio Ospedaliero San Filippo Neri—ASL Roma 1, 00161 Rome, Italy; furio.colivicchi@gmail.com; 13 UOC Cardiologia-UTIC, Ospedale Miulli, Acquaviva delle Fonti (BA), 70021 Bari, Italy; m.grimaldi@miulli.it; 14 Cardiologia 1-Emodinamica, Dipartimento Cardiotoracovascolare “A. De Gasperis”, ASST Grande Ospedale Metropolitano Niguarda, 20162 Milan, Italy; fabrizio.oliva@ospedaleniguarda.it; 15 Presidente ANMCO—Associazione Nazionale Medici Cardiologi Ospedalieri, 50121 Florence, Italy; 16 Consigliere Delegato per la Ricerca Fondazione per il Tuo Cuore-Heart Care Foundation, 50121 Florence, Italy

**Keywords:** cancer, cardioprotection, SGLT2, pathology, inflammation, metabolism, cardiotoxicity

## Abstract

Patients with active cancer and cancer survivors are at a markedly increased risk for developing cardiovascular comorbidities, including heart failure, coronary artery disease, and renal dysfunction, which are often compounded by the cardiotoxic effects of cancer therapies. This heightened cardiovascular vulnerability underscores the urgent need for effective, safe, and evidence-based cardioprotective strategies to reduce both cardiovascular morbidity and mortality. Sodium-glucose cotransporter 2 inhibitors (SGLT2is), a class of drugs originally developed for the treatment of type 2 diabetes, have demonstrated significant cardiovascular and renal benefits in high-risk populations, independent of glycemic control. Among the currently available SGLT2i, such as empagliflozin, canagliflozin, dapagliflozin, and sotagliflozin, there is growing evidence supporting their role in reducing major adverse cardiovascular events (MACEs), hospitalization for heart failure, and the progression of chronic kidney disease. Recent preclinical and clinical data suggest that SGLT2is exert cardioprotective effects through multiple mechanisms, including the modulation of inflammasome activity, specifically by reducing NLRP3 inflammasome activation and MyD88-dependent signaling, which are critical drivers of cardiac inflammation and fibrosis. Moreover, SGLT2is have been shown to enhance mitochondrial viability in cardiac cells, promoting improved cellular energy metabolism and function, thus mitigating cardiotoxicity. This narrative review critically evaluates the emerging evidence on the cardiorenal protective mechanisms of SGLT2is, with a particular focus on their potential role in cardio-oncology. We explore the common pathophysiological pathways between cardiovascular dysfunction and cancer, the molecular rationale for the use of SGLT2is in cancer patients, and the potential benefits in both primary and secondary prevention of cardiovascular toxicity related to oncological treatments. The aim is to propose a therapeutic paradigm utilizing SGLT2is to reduce cardiovascular mortality, MACE, and the burden of cardiotoxicity in high-risk oncology patients, fostering an integrated approach to cardio-oncology care.

## 1. Introduction

Cancer patients, especially cancer survivors, are exposed to several cardiometabolic diseases, including metabolic syndrome, diabetes, heart failure, and atherosclerosis. Sodium-glucose cotransporter-2 (SGLT2) inhibitors were originally developed to address type 2 diabetes mellitus (T2DM) by inhibiting glucose reabsorption in the renal proximal tubules. SGLT2is exert direct protective effects on the cardiovascular system, independently of glycemic control. Currently, SGLT2 inhibitors (SGLT2is) have been approved for the treatment of heart failure (HF) with both reduced and preserved ejection fraction. SGLT2is exert beneficial cardiorenal and systemic effects through the inhibition of NLRP-3 mediated signaling, resulting in a reduction in several cytokines and chemokines involved in cancer treatment related cardiovascular diseases. The mechanism underlying their therapeutic action explains the growing interest in cardio-oncology. In this review, we aim to analyze the potential role of SGLT2is in cardio-oncology starting with an overview on the biochemical properties of SGLT2is and their cardioprotective effects in HF. Next, we discuss of the emerging clinical evidence on the use of SGLT2is in cardio-oncology, analyzing the clinical evidence of SGLT2is in HF treatment in cancer patients and exploring their role as cardioprotective agents against cancer-treatment-related cardiac toxicity (CTRCT).

### 1.1. Biochemical Properties of SGLT2is: An Overview

Sodium-glucose co-transporter 2 inhibitors are a class of drug used primarily for managing type 2 diabetes mellitus [1]. They offer multiple benefits through various mechanisms of action [2]. However, more recent studies demonstrated its expression in several tissues, including myocardium, liver, and cancer cells [3]. In brief, this protein is responsible for reabsorbing glucose from the urine back into the bloodstream; through the inhibition of SGLT2, these drugs reduce glucose reabsorption, leading to increased glucose excretion through the urine (glycosuria) and subsequently lower blood glucose levels [4]. SGLT2is lower blood glucose levels in patients with/without type 2 diabetes independently of insulin action, which makes them effective even in patients with advanced beta-cell dysfunction [5]. Increased glucose excretion results in a loss of calories, which can contribute to weight loss by means of visceral fat reduction, associated to increased brown adipose tissue (BAT) levels [6,7]. Notably, this effect is beneficial in managing obesity, a common comorbidity in type 2 diabetes [8].

**Figure 1:**
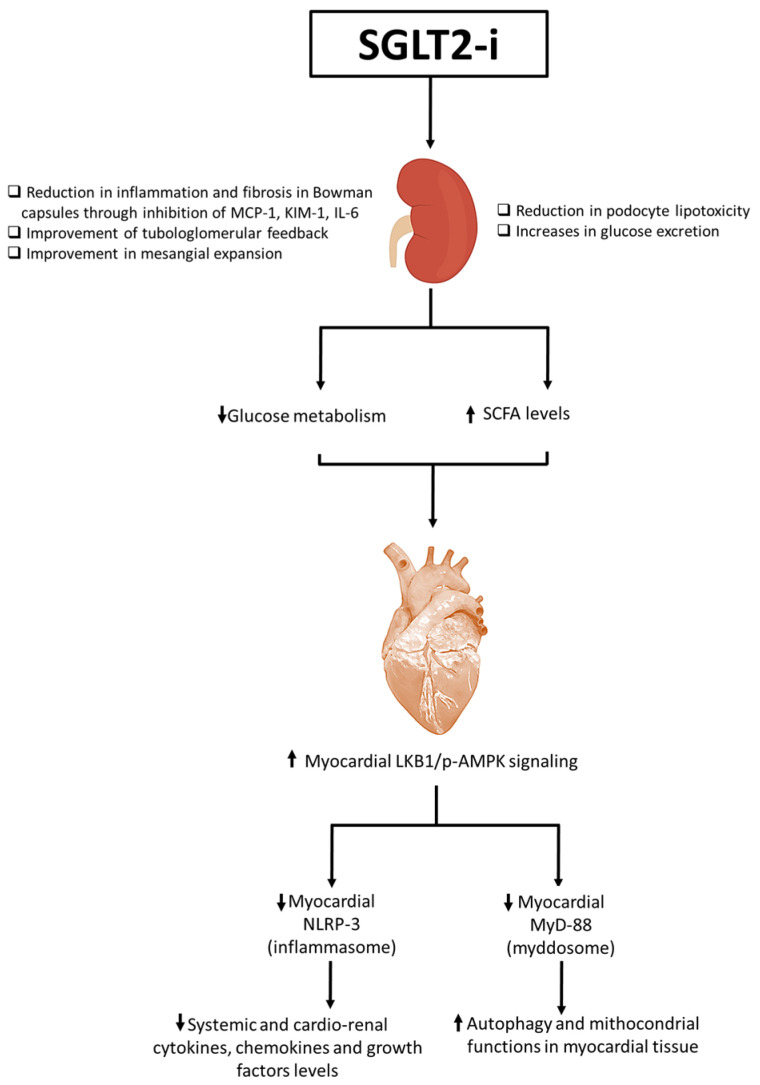
Overall illustration of SGLT2i-related cardiorenal benefits. SGLT2is increase glucose urinary excretion, improve tubule-glomerular feedback and mesangial expansion. Moreover, SGLT2is are able to reduce podocyte lipotoxicity through the inhibition of mTORC-1 pathways in podocytes and reduce inflammation and fibrosis in Bowman capsule through the inhibition of MCP-1, KIM-1, and IL-6. Beneficial renal properties reduce glucose metabolism and increase small chain fatty acid (SCFA) levels systemically and in myocardial tissue. The SCFA metabolism increases LKB-1/pAMPK signaling in myocardial cells that reduces NLRP-3 and MyD-88 pathways. The inhibition of NLP-3 and MyD88 reduces NF-kB/pro-inflammatory cytokine and chemokine pathways, resulting in autophagy and an improvement of mitochondrial functions in myocardial tissue. ⬆: increase; ⬇: decrease.

Moreover, SGLT2is have a mild diuretic effect because they increase glucose and sodium excretion; this can lead to a modest reduction in blood pressure [9]. Clinical trials have shown that SGLT2is can reduce the risk of major adverse cardiovascular events (MACE) in patients with type 2 diabetes and established cardiovascular disease; however, the clinical benefits described in recent cardiovascular outcome trials (CVOTs) will be described in paragraph 3. Moreover, SGLT2is have been associated with a slower progression of kidney disease in patients with type 2 diabetes, potentially due to their effects on reducing intraglomerular pressure and promoting natriuresis [10,11]. SGLT2is exert multiple biochemical effects: the pleiotropic effects are seen both in plasma, renal, cardiac, adipose, and hepatic tissues [12]. The effects on these organs are strictly interconnected (Figure 1). An interesting review from 2016 defines the biochemical effects of gliflozins as “a robin hood effect”, mimicking those of caloric restriction [13]. In brief, SGLT2 inhibition increases renal glucose excretion, thus reducing the availability of circulating glucose (effect mimicking caloric restriction) by increasing the glucagon–insulin ratio [14]. The increase in glucagon levels and the reduction in insulinemia increase lipolysis and reduce glycogenolysis with a consequent increase in FFA and glycerol and a reduction in pyruvate levels [15]. The resulting metabolic effect of SGLT2is is the increase in the acetyl CoA–acetoacetate–beta hydroxybutyrate pathway [16]. Peripheral tissues, especially heart, muscle, and renal cortex, will have an increase in beta-hydroxybutyrate uptake with a consequent increase in mitochondrial vitality [17].

**Figure 2:**
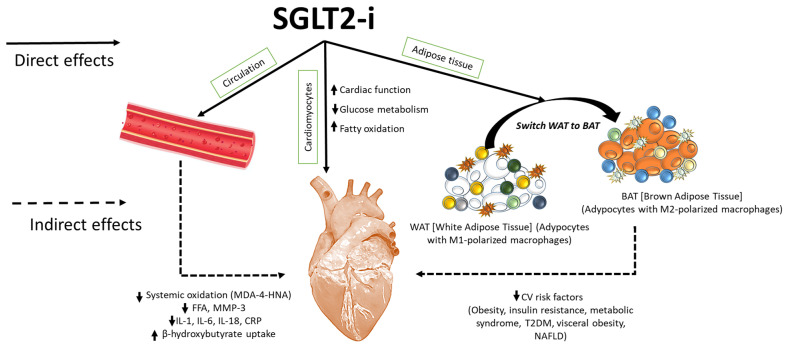
SGLT2is exert direct and indirect cardiac benefits, resulting in enhanced ejection fraction and mitochondrial viability of cardiomyocytes. SGLT2is reduce systemic levels of several cytokines, including IL-1α, IL-1β, IL-2, IL-4, IL-6, IL17-α, IFN-γ, TNF-α, G-CSF, and GM-CSF levels and hs-CRP, and reduce plasma levels of MDA and 4-HNA (lipid peroxidation products), free fatty acids, and MMP-3 levels, improving cardiac health. Moreover, SGLT2is reduce white adipose tissue content and increase brown adipose tissue, rich in M2-polarized macrophages with an-ti-inflammatory properties and anti-obesogenic activity. Switching WAT to BAT induced by SGLT2is reduces several cardiovascular risk factors, including obesity, metabolic syndrome, in-sulin resistance, type-2 diabetes mellitus (T2DM), visceral obesity, and non-alcoholic fatty liver disease (NAFLD).

Patients with T2DM are characterized by a rapid fat oxidation in heart tissue instead of glucose; there is a reduced cardiac work efficacy [18]. Biochemically, high levels of interleukin 1, 6, and 18 were found in these patients; all of this reduces myocardial contractility with a greater incidence and progression of heart failure [19]. Gliflozine treatment reduces fat oxidation and increases glucose oxidation in the cardiomyocyte, increasing β-hydroxybutyrate oxidation, and cardiac work efficacy (P/O ratio), and reducing intracellular levels of NLRP3 and pro-inflammatory cytokines [20]. This process increases myocardial contractility, reducing the incidence and progression of HF. Interestingly, researchers indicate that the hyperketonemia, such as those that prevail during treatment with SGLT2is, β-hydroxybutyrate is freely taken up by the heart (among other organs) and oxidized in preference to fatty acids [21,22]. This fuel selection improves the transduction of oxygen consumption into work efficiency at the mitochondrial level. In addition, the hemoconcentration that typically follows SGLT2is enhances oxygen release to the tissues, thereby establishing a powerful synergy with the metabolic substrate shift [23]. These mechanisms would cooperate with other SGLT2 inhibition-induced changes (enhanced diuresis and reduced blood pressure) to achieve the degree of cardioprotection revealed in the EMPA-REG OUTCOME trial [24]. More recently, other mechanisms of gliflozin-related cardiorenal benefits were analyzed, in particular, their inhibition of NLRP-3 and MyD-88 pathways in preclinical models (Figure 1). More in details, the actually known mechanisms of SGLT2i-related cardioprotective properties involves a reduction in oxidative stress and the promotion of sodium and water extraction [25]. SGLT2is are able to reduce intracellular Ca++ overload in cardiomyocytes, improving mitochondrial functions; moreover, SGLT2is are able to reduce intracellular reactive oxygen species (iROS) content and lipid peroxidation in cardiac cells, thus preventing ferroptosis [26]. Lipid peroxidation is one of the major damages induced by anthracycline therapy in cancer patients; more in detail, doxorubicin increases oxidative stress in lipid membranes, leading to the formation of MDA and 4-HNA [27]. Recent preclinical evidence clarified the anti-inflammatory properties of SGL2i due to the inhibition of NLRP-3 and MyD-88, resulting in reduced NF-kB levels and pro-inflammatory cytokines, such as IL-1β, IL-6, and IL-8 [28]. Recent research highlights the growing possibility of SGLT2is as anti-inflammatory medicines that safeguard cardiac well-being. These inhibitors present a viable option for treating cardiac tissue inflammation and lowering cardiovascular risks. As specified in Figure 1, SGLT2is increased pAMPK levels in myocardial and renal tissue, leading to beneficial mitochondrial functions and a reduction in NLRP-3 expression [29]. The stimulation of pAMPK and the downregulation of NLRP-3 reduces systemic and cardiac leptin, IL-1α, IL-1β, IL-2, IL-4, IL-6, IL17-α, IFN-γ, TNF-α, G-CSF, and GM-CSF levels [30]. Therefore, SGLT2is act as indirect inhibitors of the pro-inflammatory storm involved in CTRCD and HF in diabetic and non-diabetic patients. A significant increase in IL-10 and adiponectin levels were also seen, increasing autophagy and the ATP/ADP ratio in cardiac and renal tissues [31]. Notably, as specified in Figure 2, SGLT2is exert direct and indirect beneficial effects in myocardium. More in detail, metabolic effects described before induces a WAT to BAT switch with a M1–M2 polarization in adipocyte microenvironment [32]. Brown adipose tissues, rich in adipocytes with M2-polarized macrophages and Treg cells, change their metabolic functions to a Th1/Th17 polarization phenotype in patients with metabolic syndrome, atherosclerosis, and visceral obesity [33]. Through direct or indirect pathways, SGLT2is decreased white adipose tissue and reduced their M1 macrophages and Th1 and Th17 T cells [34]. Interestingly, inflamed white adipose tissue was associated to adipokine dysfunctions, involving high systemic levels of leptin and low levels of adiponectin and IL-10, exposing patients to a high risk of insulin resistance and metabolic syndrome [35].

### 1.2. The Revolution of SGLT2is in Cardiology

**Figure 3:**
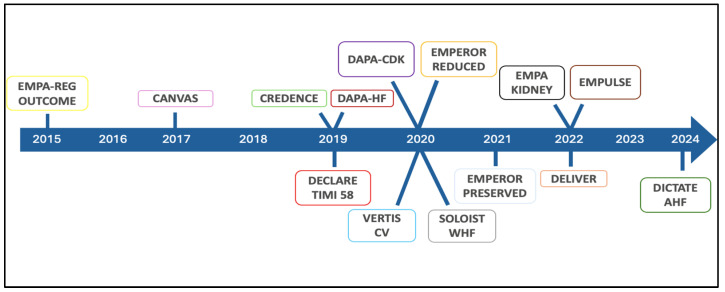
Chronology of SGLT2 inhibitor revolution in cardiology.

The studies, designed and conducted in accordance with Food and Drug Administration, that led to SGLT2i approval in patients with T2DM were designed to prove their cardiovascular (CV) safety. To pursue this aim, these studies enrolled patients with a history of major cardiovascular events (MACEs) or with prevalent and multiple risk factors [36,37]. Despite limitations related to the study design, which focused on the safety of these drugs and likely resulted in an underestimation of their true efficacy [38], these molecules have delivered exceeding expectations on CV events [36,37,39,40]. Taking a deeper dive into the results regarding the components of the primary composite endpoint in all four studies, the most impressive data concerns the extent of the reduction in the pre-defined secondary outcome of hospitalization for heart failure (HF) [36,37,39,40]. This finding has motivated the extension of clinical research particularly with two SGLT2is across the whole spectrum of patients with HF (Figure 3). At first, trials on HF with reduced ejection fraction (HFrEF) patients were conducted. The DAPA-HF (Dapagliflozin and Prevention of Adverse outcomes in Heart Failure) trial enrolled 4744 patients (58% non-diabetic) [41], and the EMPEROR-Reduced (Empagliflozin Outcome Trial in Patients with Chronic Heart Failure with Reduced Ejection Fraction) trial enrolled 3730 subjects (50.2% non-diabetic) [42]. Both studies presented consistent results for a reduction in the composite outcome of CV death or hospitalization for HF and the isolated outcome of hospitalization for HF, with reductions of 25% and 30%, respectively. CV mortality was significantly reduced in the dapagliflozin study (HR 0.82; 95% CI 0.69–0.98) along with overall mortality (HR 0.83; 95% CI 0.71–0.97), whereas it was only numerically lower in the empagliflozin study (HR 0.92; 95% CI 0.75–1.12). The disparity in results could largely be attributed to the higher frequency of hospitalizations for HF in the more severe cases of the empagliflozin study within the short follow-up period in which both studies were conducted [43].

Once the efficacy of SGLT2is in HFrEF was established, attention shifted to exploring the application of gliflozin in patients with HF with preserved ejection fraction (HFpEF) and mildly reduced ejection fraction (HFmrEF), leading to the design of the EMPEROR-Preserved study (EMPagliflozin Outcome Trial in Patients With Chronic Heart Failure with Preserved Ejection Fraction) and the DELIVER study (Dapagliflozin Evaluation to Improve the Lives of Patients with Preserved Ejection Fraction Heart Failure) [44,45]. In the EMPEROR-Preserved study, 5988 symptomatic HF patients with or without TDM2, with a left ventricular ejection fraction (LVEF) > 40%, were randomized to receive empagliflozin 10 mg or placebo [46]. The use of Empagliflozin reduced the risk of the composite end-point of CV death or hospitalization for HF (HR 0.79, CI 95% 0.69–0.90; *p* < 0.001) with the results primarily driven by a reduction in hospitalizations for HF [44]. The DELIVER study enrolled a population of 6263 symptomatic HF patients with inclusion and exclusion criteria similar to those used in the EMPEROR study, even if patients hospitalized at the time of randomization and those with a history of reduced LVEF that subsequently improved were included [47]. Patients receiving dapagliflozin reached a significant reduction in the occurrence of the primary endpoint, a composite of CV death or worsening HF events, including hospitalizations and urgent visits for worsening HF signs and symptoms, by 18% (HR 0.82, 95% CI 0.73–0.92; *p* < 0.001) [45]. Similar to the results in patients with HFrEF, treatment with empagliflozin and dapagliflozin was well tolerated and safe in both the EMPER-OR-Preserved and DELIVER studies [48]. Another key finding from these studies, which highlights the revolutionary impact of these drugs, is the significant reduction in the loss of renal function demonstrated in patients receiving SGLT2is [49]. This renal-protective effect has been supposed to contribute to the overall effectiveness of SGLT2is in HF patients and has sparked interest in their use also in patients with chronic kidney disease (CKD). Particularly, the DAPA-CKD trial (Dapagliflozin in Patients with Chronic Kidney Disease) enrolled 4304 subjects with an estimated glomerular filtration rate (eGFR) between 25 and 75 mL/min/1.73 m^2^ and an albumin-to-creatinine ratio (UACR) between 200 and 5000, intending to explore the occurrence of a composite primary end-point of sustained decline in the eGFR by at least 50% for end-stage kidney disease (ESKD) and death from renal or CV causes. After a mean follow-up of 2.4 years, a primary outcome occurred in 9.2% of the participants in the dapagliflozin group and 14.5% of patients in the placebo group (HR 0.61; 95% CI 0.51–0.72; *p* < 0.001; NNT: 19; 95% CI 15–27) [50]. The EMPA-KIDNEY trial (Empagliflozin in Patients with Chronic Kidney Disease) enrolled 6609 patients with and without T2DM and with CKD (eGFR 20–45 mL/min/1.73 m^2^ or eGFR 45–90 mL/min/1.73 m^2^ and UACR ≥ 200 mg/g), exploring a primary outcome defined as the progression of kidney disease (an occurrence of ESKD, eGFR < 10 mL/min/1.73 m^2^, reduction in eGFR ≥ 40% from baseline, and death from renal causes) or CV death. Patients treated with empagliflozin demonstrated a 28% reduction in the risk of the primary outcome (HR 0.72 [0.64–0.82], *p* < 0.001), primarily driven by the effect on the progression of kidney disease (HR 0.71) [16]. Interestingly, this trial broadens the characteristics of the population that benefits from such nephroprotection, including over 50% of non-diabetic patients (vs. 30% in DAPA-CKD), 35% of patients with an eGFR < 30 mL/min/1.73 m^2^ (vs. 4% and 15%), and 50% of patients with microalbuminuria (vs. 11% and 10%), of which 20% had normoalbuminuria [51]. The demonstration of a similar impact of these molecules on comorbidities widely prevalent in the oncological population underscores the absolute necessity of exploring their use in the field of cardio-oncology.

### 1.3. SGLT2is in the Treatment of Cancer Patients with Heart Failure or Cardiac Toxicity

SGLT2is have demonstrated improvement in cardiac outcomes in patients with heart failure (HF), independent of diabetes status and left ventricular ejection fraction (LVEF) [52,53]. The updated 2022 American College of Cardiology, American Heart Association, and Heart Failure Society of America clinical practice guidelines and the 2021 European Society of Cardiology recommend the use of SGLT2is as part of guideline-directed medical therapy (GDMT) for HFrEF, heart failure with midrange ejection fraction (HFmrEF), and HFpEF [54,55]. This suggests their potential role in cardio-oncology by improving cardiac outcomes in patients with cancer treated with cardiotoxic cancer therapies and HF.

After the recurrence of the primary cancer, cardiovascular disease emerges as the second leading cause of morbidity and mortality in cancer survivors [56]. Anticancer treatments are associated with serious cardiovascular adverse events, including HF, hypertension, arrhythmias, and coronary artery disease. Among these treatments, anthracyclines, widely used in the treatment of patients with various cancer types, have the highest risk for cardiotoxicity [57]. Radiotherapy was associated with dose-dependent cardiac injury, including valvular disorders or ischemia or pericardial injury [58]. Trastuzumab and anti-HER2 therapies caused cardiotoxicity from asymptomatic decline in left ventricular (LV) ejection fraction to symptomatic HF, an effect that can worsen when combined with anthracyclines [59]. Tyrosine kinase inhibitors such as sunitinib can have also cardiovascular adverse effects, including hypertension and HF [60]. Carfilzomib was associated with hypertension, HF, and ischemia in more than 18% of patients [61]. Therefore, it is critical to investigate if SGLT2is can reverse the cardiovascular complications arising from cancer treatments. Although SGLT2is have demonstrated efficacy in the treatment of patients with ischemic and nonischemic cardiomyopathy and HF, patients with cancer are usually excluded from clinical trials. Hence, the efficacy of SGLT2is remains understudied in patients with cancer-therapy-related cardiac dysfunction (CTRCD)/HF.

Recently a case series [62] of consecutive patients diagnosed with CTRCD/HF who were given SGLT2is in addition to guideline-directed medical treatment (GDMT) was published. Seven patients with anthracycline-related cardiac dysfunction were clinically and echocardiographically evaluated before and after the introduction of SGLT2is. After a median period of 24 weeks with uninterrupted treatment, a significant clinical improvement was observed with at least one New York Heart Association Functional Class (NHYA FC) improvement in all patients (median NYHA FC: I vs. III, *p* < 0.010). A left ventricular reserve remodeling (median left ventricular end diastolic volume indexed: 53 vs. 82.5 mL/m^2^, *p* = 0.018; median left ventricular ejection fraction: 50% vs. 40%, *p* = 0.17) was also observed; no cases of discontinuation or relevant side effects were observed (Table 1).

A recent retrospective study assessed the efficacy of SGLT2is in patients experiencing cardiac dysfunction due to cancer therapy, including anthracyclines, alkylating agents, antimetabolites, monoclonal antibodies, tyrosine kinase inhibitors, proteasome inhibitors, and radiation therapy [63]. The study focused on patients 18 years of age with histories of T2DM, cancer, and exposure to potentially cardiotoxic antineoplastic therapies, with subsequent diagnoses of cardiomyopathy or HF between 2013 and 2020. The study cohort included 1280 patients experiencing cardiac dysfunction or HF related to cancer therapy (640 in each group, categorized on the basis of SGLT2i use). Hematologic cancer was the most common, followed by gastrointestinal cancer, breast cancer, and lymphoma. Over a 2-year follow-up period, patients on SGLT2is in addition to conventional guideline-directed medical therapy had a lower risk of acute HF exacerbation (OR: 0.483 [95% CI: 0.36–0.65]; *p* < 0.001) and all-cause mortality (OR: 0.296 [95% CI: 0.22–0.40]; *p* = 0.001). All-cause hospitalizations or emergency department visits (OR: 0.479; 95% CI: 0.383–0.599; *p* < 0.001), atrial fibrillation/flutter (OR: 0.397 [95% CI: 0.213–0.737]; *p* = 0.003), acute kidney injury (OR: 0.486 [95% CI: 0.382–0.619]; *p* < 0.001), and need for renal replacement therapy (OR: 0.398 [95% CI: 0.189–0.839]; *p* = 0.012) were also less frequent in patients on SGLT2is. From a safety perspective, this study shows that SGLT2i use is safe in patients with CTRCD. Recently, a prospective/retrospective observational trial on the use of SGLT2is in 83 active cancer patients has been presented. The study confirmed the safety and effectiveness of gliflozins in this patient category with no new safety warning, hence supporting the use of these drugs in active cancer patients [64] (Table 1).

**Table 1:** Clinical studies demonstrating cardioprotective effects of SGLT2 inhibitors in cancer-therapy-related cardiac dysfunction.

Study	Design	Population	Cancer Therapy	SGLT2i Intervention	Key Findings
**Case Series [62]**	Prospective, single-arm	7 patients with anthracycline-induced HF (CTRCD)	Anthracyclines	SGLT2is (empagliflozin or dapagliflozin) added to standard GDMT	–NYHA class improved in all patients (median III → I, *p* < 0.010) –LVEF improved (40% → 50%, *p* = 0.17) –LVEDVi reduced (82.5 → 53 mL/m^2^, *p* = 0.018) –No treatment discontinuations or major adverse effects
**Retrospective Cohort Study [63]**	Retrospective matched cohort (*n* = 1280)	Patients ≥ 18 years with cancer, T2DM, and HF post-cardiotoxic therapy	Anthracyclines, alkylating agents, antimetabolites, anti-HER2, TKIs, proteasome inhibitors, radiation	SGLT2is + GDMT vs. GDMT alone	–Reduced HF exacerbations: OR 0.483 (95% CI: 0.36–0.65, *p* < 0.001) –Lower all-cause mortality: OR 0.296 (95% CI: 0.22–0.40, *p* = 0.001) –Fewer hospitalizations and ED visits: OR 0.479 (95% CI: 0.383–0.599, *p* < 0.001) –Reduced atrial fibrillation, AKI, and RRT need
**Observational Trial [64]**	Mixed prospective/retrospective	83 patients with active cancer (various types)	Various antineoplastic therapies	SGLT2is (unspecified type) + GDMT	–SGLT2is well tolerated with no new safety concerns –Confirmed effectiveness and clinical stability in oncology patients

Abbreviations: CTRCD = cancer-therapy-related cardiac dysfunction; HF = heart failure; LVEF = left ventricular ejection fraction; LVEDVi = LV end-diastolic volume indexed; NYHA = New York Heart Association; GDMT = guideline-directed medical therapy; OR = odds ratio; ED = emergency department; AKI = acute kidney injury; RRT = renal replacement therapy; T2DM = type 2 diabetes mellitus; TKIs = tyrosine kinase inhibitors.

### 1.4. Safety Considerations and Contraindications in Oncology Settings

While SGLT2is show promise in mitigating cardiovascular and renal complications in cancer patients, their use in this population warrants careful consideration of safety and potential contraindications, particularly in immunocompromised, catabolic, or dehydrated patients. The most frequently reported adverse effects in non-oncological populations include genitourinary infections, dehydration, and electrolyte disturbances [62].

These concerns are amplified in cancer patients, especially those receiving chemotherapy or immunosuppressive therapies, who are already at heightened risk for infections and fluid imbalance. Genitourinary infections, such as urinary tract infections and genital mycotic infections, are known side effects of SGLT2is, and the use of these agents in cancer patients, particularly those with indwelling catheters or who are undergoing treatments that increase susceptibility to infections, requires caution [63].

Furthermore, the risk of euglycemic diabetic ketoacidosis (DKA), although relatively rare, is an established safety concern with SGLT2is. While DKA is typically associated with hyperglycemia, SGLT2is can induce ketoacidosis in patients with normal glucose levels, especially in those who are under metabolic stress, such as in the setting of cancer, infection, or dehydration.

The immunocompromised status of many cancer patients may predispose them to this potentially life-threatening complication, particularly in the absence of early recognition. Dehydration and fluid imbalance are also major concerns as SGLT2is have a diuretic effect that can exacerbate pre-existing fluid deficits in cancer patients, leading to hypotension, acute kidney injury, or electrolyte disturbances such as hyponatremia and hyperkalemia [64]. These risks are particularly pertinent in the context of chemotherapy-induced toxicity, which can further complicate fluid and electrolyte homeostasis. Given the complexity of managing cancer patients with these risk factors, careful monitoring of renal function, electrolytes, and hydration status is critical when considering SGLT2i therapy. Therefore, while SGLT2is may offer significant therapeutic benefits, their use in oncology should be approached with caution, with appropriate patient selection, close monitoring, and a thorough risk–benefit assessment. Further clinical research is urgently needed to establish clear safety guidelines and identify any cancer-specific contraindications for their use in some high-risk patients [62,63].

### 1.5. Cardioprotective Effects of SGLT2 Inhibitors in Cancer-Therapy-Related Cardiac Dysfunction (CTRCD): Preclinical and Clinical Evidence

A growing body of preclinical data supports the cardioprotective potential of SGLT2 inhibitors in models of CTRCD, in particular, of anthracycline-induced cardiotoxicity [25]. Anthracyclines induce myocardial injury via multiple pathways, including reactive oxygen species (ROS) generation, mitochondrial dysfunction, pro-inflammatory cytokine release, and apoptotic signaling [26].

SGLT2is have demonstrated off-target effects that counteract several of these cardiotoxic mechanisms. In murine models, empagliflozin and dapagliflozin have been shown to preserve LVEF, reduce myocardial fibrosis, attenuate lipid peroxidation, and inhibit caspase-3-dependent apoptosis in hearts exposed to doxorubicin. Mechanistically, these benefits are thought to arise from improved mitochondrial bioenergetics, the upregulation of antioxidant defense systems (e.g., Nrf2/HO-1 pathway), the modulation of autophagy, and the suppression of NLRP3 inflammasome activation.

Notably, these effects are observed independently of glycemic status, suggesting that the cardioprotective properties of SGLT2 inhibitors extend beyond diabetic models and may be applicable to non-diabetic cancer patients [25,26,27]. In addition, empagliflozin has been shown to enhance myocardial ATP production and reduce cytosolic sodium and calcium overload via the inhibition of the cardiac Na⁺/H⁺ exchanger, a mechanism implicated in doxorubicin-induced cardiotoxicity [26].

While clinical evidence remains limited, emerging data suggest a potential role for SGLT2is in mitigating cancer-therapy-related cardiac dysfunction in humans. Retrospective analyses and small prospective observational studies have reported improved cardiac outcomes in patients receiving SGLT2 inhibitors during or after anthracycline therapy [58].

For example, in a pilot study of patients with breast cancer undergoing doxorubicin-based chemotherapy, those concurrently treated with empagliflozin exhibited smaller declines in LVEF and lower elevations in high-sensitivity troponin I compared to matched controls [61,62]. Another real-world study reported a reduced incidence of heart failure hospitalizations in diabetic cancer patients exposed to SGLT2 inhibitors during chemotherapy, though causality remains unproven [56].

Importantly, these findings are consistent with the broader cardiovascular benefits observed in large heart failure trials (e.g., DAPA-HF and EMPEROR-Reduced), which lend further mechanistic plausibility to their application in cardio-oncology [38,41,42,43].

However, prospective randomized controlled trials specifically targeting patients at risk for or experiencing CTRCD are currently lacking. Several ongoing trials, such as the EMPACT trial (Empagliflozin for Prevention of Anthracycline-Induced Cardiotoxicity; NCT05271162), aim to clarify the safety, efficacy, and optimal timing of SGLT2 inhibitor use in oncology populations [38,41,42,43,44,45].

### 1.6. Clinical Considerations for the Use of SGLT2 Inhibitors in Cardio-Oncology

The incorporation of SGLT2is into cardio-oncology care requires a nuanced understanding of patient-specific cardiovascular risk profiles, cancer treatment regimens, and comorbid conditions [42]. Among the currently approved agents, empagliflozin (10 mg once daily) and dapagliflozin (10 mg once daily) have the most robust cardiovascular and renal outcome data and are approved for use in patients with heart failure with HFrEF, CKD, and T2DM, all common in patients with cancer. In the cardio-oncology setting, these agents are best considered for

- Patients with established HFrEF (LVEF ≤ 40%), where they confer well-documented reductions in cardiovascular mortality and heart failure hospitalization;
- Cancer patients receiving high-risk cardiotoxic therapies—such as high cumulative-dose anthracyclines (>250–300 mg/m^2^), HER2-targeted therapies (e.g., trastuzumab), or thoracic radiation—who are at an elevated risk for developing CTRCD;
- Patients with early signs of cardiac dysfunction, including abnormal global longitudinal strain (GLS), rising high-sensitivity troponin or NT-proBNP, or asymptomatic declines in LVEF (i.e., Stage B heart failure).

Optimal timing of initiation should align with a proactive risk-based strategy, ideally before or early during the course of cardiotoxic chemotherapy, particularly in those with underlying CV risk factors (e.g., hypertension, diabetes, CKD, or prior cardiovascular disease). In patients without overt HFrEF, SGLT2 inhibitors may be considered as cardioprotective adjuncts if early functional or biomarker-based evidence of myocardial stress emerges, though this remains investigational. For cancer patients already diagnosed with HFrEF, initiation can occur either in the inpatient or outpatient setting, with no titration needed due to the fixed-dose regimen. The choice between empagliflozin and dapagliflozin can be individualized based on availability, insurance coverage, and familiarity, as both show comparable efficacy profiles.

Notably, renal function should be assessed prior to initiation; both agents are approved for use down to eGFR thresholds of 20–25 mL/min/1.73 m^2^, although diuretic effects may be attenuated at lower GFR [62].

Patients should be euvolemic at baseline, and temporary interruption is recommended during periods of acute illness, dehydration, or reduced oral intake (e.g., during high-dose chemotherapy or gastrointestinal toxicities). Moreover, SGLT2 inhibitors should be used cautiously in patients at risk for euglycemic diabetic ketoacidosis (euDKA) or those with active genitourinary infections [60,61,62,63,64,65].

Therefore, a close coordination between oncology, cardiology, and nephrology is critical to ensure safe implementation.

Though randomized controlled trial data in cancer populations are still pending, emerging real-world evidence and mechanistic plausibility support their use as preventive or therapeutic agents in carefully selected cardio-oncology patients. Ongoing trials, including EMPACT (NCT05271162) and DAPA-ONCO (NCT05695403), are expected to provide needed clarity on the role of these agents in preventing or attenuating anthracycline-induced cardiotoxicity. On the basis of these considerations, here is presented a proposed clinical algorithm for the use of SGLT2is in cancer patients (Table 2).

In brief, this stepwise clinical algorithm is designed to guide the practical use of SGLT2 inhibitors in cancer patients at risk for or affected by CTRCD. It integrates cardiovascular risk stratification, cancer treatment type, early indicators of cardiac injury, and patient-specific considerations such as renal function, volume status, and glycemic control [27]. Therefore, SGLT2 inhibitors should be considered in cancer patients with established HFrEF, as well as those undergoing high-risk cardiotoxic therapies or exhibiting early signs of cardiac dysfunction (e.g., abnormal GLS, rising troponin or NT-proBNP) [63,66].

Appropriate patient selection, the timing of initiation, and close multidisciplinary coordination are essential to optimize cardioprotective benefit while minimizing potential adverse effects. This algorithm is intended to support individualized, evidence-informed decision-making in the cardio-oncology setting.

## 2. Discussion

Cardiotoxicity remains a major clinical challenge in the management of cancer patients, representing a critical intersection between oncology and cardiovascular medicine. With the increasing efficacy of modern oncologic therapies, long-term survivorship has improved substantially [3]; however, this has been paralleled by a rising burden of cardiovascular complications, including heart failure, arrhythmias, and ischemic events. Among these, MACE and CV mortality are particularly concerning, often leading to premature treatment discontinuation and reduced oncologic outcomes [6]. Anthracyclines, HER2-targeted therapies, immune checkpoint inhibitors, and newer tyrosine kinase inhibitors have all been implicated in various forms of cardiotoxicity, through mechanisms ranging from direct myocardial injury to endothelial dysfunction and inflammation. The complex and multifactorial nature of cancer-therapy-related cardiac dysfunction (CTRCD) underscores the pressing need for proactive, mechanism-targeted cardioprotective strategies [3,4,5,6,7,8]. Despite existing surveillance protocols and supportive therapies, there is a clear unmet need for pharmacologic interventions that can both prevent and mitigate CV injury without compromising antitumor efficacy. In this context, SGLT2is have emerged as promising agents, given their robust cardioprotective effects demonstrated across multiple large-scale cardiovascular outcome trials in patients with heart failure, diabetes, and chronic kidney disease [10,11,12,13]. Notably, recent studies have highlighted an emerging role for SGLT2is in modulating erythropoiesis, a critical process in the regulation of red blood cell production. SGLT2is, by reducing renal glucose reabsorption and lowering serum glucose levels, indirectly enhance erythropoiesis through the activation of hypoxia-inducible factor (HIF)-1α [66].

The reduction in renal glucose reabsorption and the subsequent mild diuretic effect of SGLT2is lead to a relative state of hypoxia in the renal cortex, stimulating the production of erythropoietin (EPO). Erythropoietin, in turn, promotes the proliferation and differentiation of erythroid progenitor cells in the bone marrow, thereby increasing red blood cell production [20,66]. In addition to this indirect effect, SGLT2is have been shown to attenuate inflammation and improve oxidative stress, two factors that often impair erythropoiesis in various disease states, including chronic kidney disease and cancer. Notably, the ability of SGLT2is to modulate erythropoiesis is of particular interest in oncology patients, where anemia is a common and debilitating complication. By promoting erythropoiesis, SGLT2is may improve anemia-associated fatigue and the overall quality of life in these high-risk populations [67]. These effects underscore the pleiotropic benefits of SGLT2 inhibitors, which extend beyond their well-established cardiorenal protective roles to encompass the modulation of hematopoiesis, providing a novel therapeutic approach in the management of anemia in cancer patients [67].

However, the potential role of SGLT2is in cardio-oncology, however, remains underexplored and warrants critical evaluation as a novel avenue to reduce MACE, CV mortality, and treatment-limiting cardiac complications in this high-risk population.

**Figure 4:**
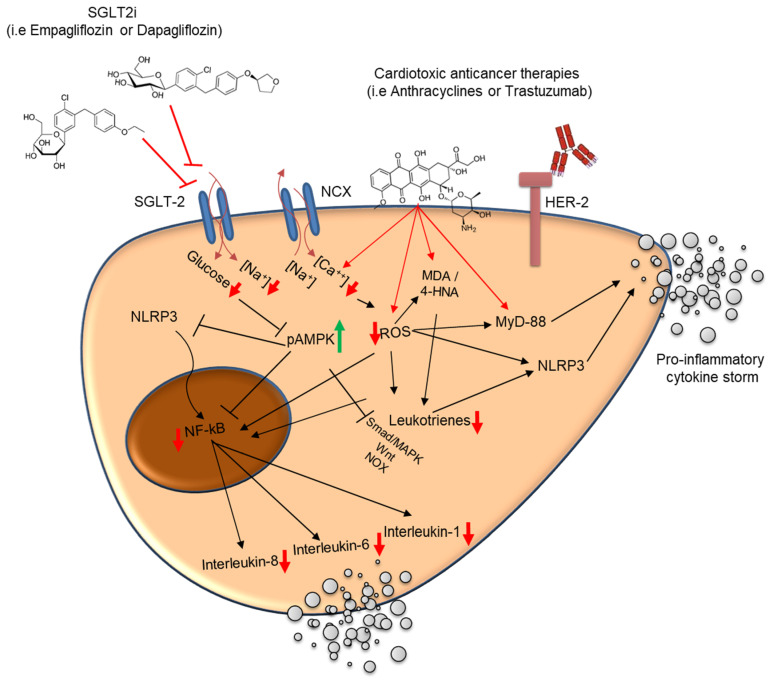
Proposed molecular mechanisms underlying the cardioprotective effects of SGLT2 inhibitors against anticancer-therapy-induced cardiotoxicity. Antineoplastic agents like anthracyclines increase intracellular Ca^2+^ concentrations, leading to mitochondrial dysfunction and elevated reactive oxygen species (ROS) production. ROS accumulation induces lipid peroxidation products (MDA and 4-HNA) and activates MyD-88 and the NLRP3 inflammasome, culminating in nuclear factor-kappa B (NF-κB) activation and transcription of pro-inflammatory cytokines (IL-1β, IL-6, and IL-8). These processes contribute to maladaptive myocardial inflammation and remodeling. SGLT2 inhibitors attenuate intracellular Na^+^ and Ca^2+^ overload, reduce oxidative stress, and inhibit MyD-88/NLRP3-mediated inflammatory pathways. Furthermore, SGLT2is activate AMPK (pAMPK), which enhances mitochondrial viability and suppresses NF-κB signaling. The combined effect is a reduction in pro-inflammatory cytokine release and oxidative injury, promoting cardiomyocyte survival and limiting cancer-therapy-related cardiac dysfunction. ⬆: increase; ⬇: decrease.

Currently, there is a lack of clinical evidence in the role of SGLT2is to prevent cardiac dysfunction induced by anti-cancer therapies. The preclinical evidence and biochemical properties that could support their cardioprotective role have been previously reported [65,66,68,69]. As shown in Figure 4, SGLT2 inhibitors exert cardioprotective effects in the setting of anticancer-therapy-induced cardiotoxicity by modulating several interrelated molecular pathways implicated in oxidative stress, inflammation, and intracellular ionic homeostasis. Cardiotoxic agents, such as anthracyclines or HER-2 blocking agents, promote intracellular accumulation of calcium (Ca^2+^), leading to mitochondrial dysfunction and excessive production of reactive oxygen species (ROS). This oxidative environment facilitates lipid peroxidation (e.g., the generation of malondialdehyde [MDA] and 4-hydroxynonenal [4-HNA]), which subsequently activates MyD-88 and the NLRP3 inflammasome pathway. These cascades converge on nuclear factor-kappa B (NF-κB), a transcription factor that orchestrates the expression of pro-inflammatory cytokines, including interleukin (IL)-1β, IL-6, and IL-8, thereby amplifying myocardial inflammation and injury. SGLT2 inhibitors counteract these deleterious effects by limiting Na⁺ and Ca^2+^ influx into cardiomyocytes, thereby attenuating mitochondrial overload and reducing ROS generation. Additionally, these agents upregulate phosphorylated AMPK (pAMPK), a key metabolic regulator that preserves mitochondrial viability, inhibits NF-κB activation, and promotes cellular resilience under stress [26,27].

Through both direct and indirect inhibition of NLRP3 and MyD-88 signaling, SGLT2 inhibitors dampen the production of pro-inflammatory cytokines and mitigate leukotriene-mediated inflammatory responses [26]. The net result is a reduction in cardiac inflammation, the preservation of myocardial structure and function, and the attenuation of the remodeling processes associated with cancer-therapy-related cardiac dysfunction [27].

Clinically, Sabatino et al. [66] evaluated the impact of empagliflozin on anthracycline-induced cardiotoxicity and studied the underlying molecular mechanisms on adult mammalian hearts. In particular, they spotlighted the involvement of SGLT1 and SGLT2 receptors and the stress signaling pathways, including extracellular signal-regulated kinase (ERK), Janus kinase (JNK), and mitogen-activated protein kinases (MAPKs) in a murine model of cardiotoxicity induced by doxorubicin. In this study, empagliflozin demonstrated an ability to improve left ventricle ejection fraction, fraction shortening, longitudinal strain, and circumferential strain. Moreover, they saw in the SGLT2 inhibitor group a lower degree of myocardial fibrosis, a reduction in the disarray of myocardial fibers, and a reduction in wavy myocardial fibers. Immunohistochemistry confirmed, furthermore, the expression of SGLT2 in mice hearts. SGLT2 expression showed a 75% reduction in DOX mice, compared to baseline (*p* < 0.001). This effect was significantly attenuated in EMPA + DOX mice (*p* = 0.263). Sabatino et al. [66] studied a signaling pathway considered to be potentially involved in the chemotherapy-induced cardiotoxicity. Indeed, ERK activity was significantly increased in DOX mice compared to controls (*p* = 0.018). Interestingly, treatment with empagliflozin (EMPA + DOX) was associated with a 36%-lower level of ERK activity compared with doxorubicin alone (DOX) (*p* = 0.038). To be conclusive, the studied mentioned above demonstrated that treatment with empagliflozin prevented the reduction in cardiac systolic function induced by a cardiotoxic anthracycline in a model of non-diabetic mice [66]. Quagliariello et al. [26] performed preliminary cellular studies on mouse cardiomyocytes (HL-1 cell line) exposed to doxorubicin alone or combined to EMPA. Cardiomyocytes exposed to doxorubicin increased the intracellular Ca2+ content and expression of several pro-inflammatory markers associated to cell death; co-incubation with EMPA reduced significantly the magnitude of the effects. In the preclinical study, EMPA increased the ejection fraction compared to the DOXO groups (*p* < 0.05), prevented the reduction in radial and longitudinal strain after 10 days of treatment with doxorubicin (RS) 30.3% in EMPA-DOXO vs. 15.7% in DOXO mice; the LS was −17% in EMPA-DOXO vs. −11.7% in DOXO mice (*p* < 0.001 for both). Significant reductions in ferroptosis, xanthine oxidase expression, cardiac fibrosis, and apoptosis in EMPA associated with DOXO were also seen. A reduced expression of pro-inflammatory cytokines, NLRP3, MyD88, and NF-kB in the heart, liver, and kidneys was also seen in DOXO-EMPA group compared to DOXO (*p* < 0.001). These findings provide the proof of concept for translational studies designed for the employment of these medications to prevent cardiotoxicity induced by chemotherapy.

Gongora et al. [67] conducted a retrospective study to test the cardiac efficacy and overall safety of SGLT2is in patients treated with anthracyclines. They identified 3033 patients with diabetes mellitus (DM) and cancer who were treated with anthracyclines. The primary cardiac outcome was a composite of cardiac events (heart failure incidence, heart failure admissions, new cardiomyopathy [>10% decline in ejection fraction to <53%), and clinically significant arrhythmias). The primary safety outcome was overall mortality. There were 20 cardiac events over a median follow-up period of 1.5 years. The cardiac event incidence was lower among case patients in comparison to control participants (3% vs. 20%; *p* = 0.025). Case patients also experienced lower overall mortality when com-pared with control participants (9% vs. 43%; *p* < 0.001) and a lower composite of sepsis and neutropenic fever (16% vs. 40%; *p* = 0.013). The authors conclude that SGLT2is were associated with a lower rate of cardiac events among patients with cancer and DM who were treated with anthracyclines. Additionally, SGLT2is appeared to be safe. Recently, Bhatti et al. [70] conducted a retrospective analysis of patients database with T2DM, cancer, exposure to cardiotoxic therapies, and no prior documented history of cardiomyopathy or heart failure. This study sought to determine whether SGLT2i use is associated with a lower incidence of CTRCD. The study included 8675 propensity-matched patients in each cohort (mean age ¼ were 65 years, 42% females, 71% White, 19% gastrointestinal malignancy, and 25% anthracyclines). Patients prescribed SGLT2is had a lower risk of developing CTRCD (HR: 0.76: 95% CI: 0.69–0.84). SGLT2is also reduced heart failure exacerbations (HR: 0.81; 95%CI: 0.72–0.90), all-cause mortality (HR: 0.67; 95% CI: 0.61–0.74), and all-cause hospitalizations/emergency department visits (HR: 0.93; 95% CI: 0.89–0.97). Subgroup analyses also demonstrated a reduced CTRCD risk across various classes of cancer therapies in patients prescribed SGLT2is. These data support the conducting of a randomized clinical trials testing SGLT2is in patients with cancers undergoing cardiotoxic therapies to reduce the risk of cardiotoxicity. To this end, there are three ongoing trials testing the cardioprotective role of SGLT2is: the EMPACT (Empagliflozin in the Prevention of Cardiotoxicity in Cancer Patients Undergoing Chemotherapy Based on Anthracyclines) trial, a randomized, multicenter, placebo-controlled, double blind clinical trial (NCT05271162) with aims to determine the efficacy of empagliflozin in preventing LV systolic dysfunction in patients with cancer exposed to high cumulative doses of anthracyclines; the PROTECT (Potential protective ROle of SGLT2 inhibitors for chemoThErapy-induced CardioToxicity) phase II “proof of concept”, multicenter, randomized, open label, parallel-groups study (NCT06341842) with the aim to investigate the role of dapagliflozin in preventing chemotherapy-induced cardiotoxicity in participants with breast cancer treated with (neo-)adjuvant anthracycline-based chemotherapy +/− anti-HER2 therapies; the PROTECTAA Trial (CardioPROTECTion with Dapagliflozin in Breast Cancer Patients Treated with AnthrAcycline) a multicenter, randomized, double-blind, placebo-controlled phase III study (NCT06304857), evaluating the effect of dapagliflozin on prevention of cardiotoxicity in breast cancer patients undergoing anthracycline-based chemotherapy.

However, several limitations of the current review should be noted. First, the lack of formal systematic review processes and quality assessment tools may introduce selection bias. Additionally, as most studies in this area are still in early stages, the evidence base is limited, with a predominance of observational data and small-scale trials. Therefore, our conclusions should be viewed as preliminary, and further large-scale, well-controlled studies are needed to validate the findings and address the unanswered questions in this field.

Notably, while the available evidence on the cardiorenal protective effects of SGLT2 inhibitors in oncology is promising, it remains preliminary, and caution is warranted when interpreting these findings. Current studies have highlighted potential benefits, including the modulation of inflammatory pathways, mitochondrial protection, and improvement of erythropoiesis, but these effects must be confirmed in larger, rigorously designed clinical trials. The current body of evidence is currently in progress, and further research is essential to establish their true cardioprotective role in this high-risk population. Prospective, large-scale clinical trials are needed to validate the observed benefits, evaluate long-term safety, and clarify the impact of SGLT2 inhibitors on cardiovascular outcomes in patients undergoing cancer therapy.

These trials should aim to elucidate not only the efficacy but also the optimal therapeutic strategies, including patient selection criteria, treatment duration, and combination with other therapeutic modalities. Until these critical data are available, the use of SGLT2 inhibitors in oncology should be considered as an area of interest for future research rather than as an established clinical practice.

## 3. Methods

A narrative search of the Medline and EMBASE databases was conducted to identify relevant papers reporting original research, including randomized controlled trials (RCTs), cohort studies, and meta-analyses, on the role and cardiovascular effects of sodium-glucose co-transporter 2 inhibitors (SGLT2is, including gliflozins) in patients with cancer or cardiovascular diseases over the past 10 years. The search strategy was designed to capture evidence across cardiology and cardio-oncology settings. Clinical studies were included if they met the following criteria:

- Published in English with an available abstract;
- Addressed at least one of the following topics: SGLT2 inhibitors, gliflozin, cardiotoxicity, cardiovascular diseases, cardio-oncology, cancer, cardioprotection, or cardiometabolic health.

Studies were excluded if they were reviews, case reports, lacked endpoints related to cardiovascular toxicity or cardioprotection, or were conducted exclusively in surgical or perioperative settings. We qualitatively synthesized the findings from the selected studies to provide a comprehensive overview of the emerging role of SGLT2is in cardio-oncology. Boolean operators (AND, OR) were employed in the search strategy without truncation (*) to optimize sensitivity and relevance. The search terms were adapted for each database, and the exact strings are reported in Table 3. The databases were last accessed on 27 March 2025. In addition to the search criteria mentioned, a comprehensive review of study quality was conducted. While a formal systematic quality assessment tool was not applied, the selected studies were evaluated based on the robustness of their design, sample size, and relevance to the review topic. We aimed to include high-quality studies, including those with rigorous statistical analysis and well-defined outcomes related to cardiovascular effects and cardiotoxicity. However, given the nature of the narrative review, studies with varying degrees of methodological rigor were included to ensure a broad overview of the current landscape. As a narrative review, we did not perform meta-analysis or quantitative synthesis of data. Instead, we qualitatively synthesized the findings from the selected studies, highlighting key trends and areas of consensus, as well as gaps in the literature. Acknowledging the diversity in study design and methodologies, we refrained from making direct comparisons between studies or drawing definitive conclusions regarding treatment efficacy.

## 4. Conclusions

In conclusion, SGLT2is appear promising in cardio-oncology considering their beneficial and safe effects in the management of HF, T2DM, and chronic kidney disease and preclinical evidence in cardio-oncology. The overall picture of the review encourages the use of SGLT2is in cancer patients as a therapeutic option for treatment of cancer therapy–related cardiac dysfunction/HF; although further research and prospective studies are definitely needed to evaluate the efficacy of SGLT2is in this population. Therefore, randomized and prospective studies in patients under treatment with cardiotoxic cancer treatments are certainly needed to investigate the safety and efficacy of SGLT2is in primary prevention.

## Figures and Tables

**Table 2:** Proposed clinical algorithm for the use of SGLT2 inhibitors in cancer patients.

Step	Assessment Domain	Clinical Consideration	Recommended Action
**1**	**Baseline Cardiovascular Risk**	History of HfrEF *, ASCVD, hypertension, diabetes, CKD, prior cardiotoxic therapy	Consider SGLT2is in patients with established HFrEF (LVEF ≤ 40%) or high baseline CV risk
**2**	**Planned or Ongoing Cancer Therapy**	Anthracyclines (>250 mg/m^2^), HER2 inhibitors, thoracic radiation, TKi	High-risk regimens may benefit from early prophylactic SGLT2i initiation if no contraindications
**3**	**Subclinical Cardiac Dysfunction**	Elevated troponin, NT-proBNP, abnormal GLS, asymptomatic ↓ LVEF	Consider SGLT2is for cardioprotection in patients showing early cardiac injury markers, even in absence of symptoms
**4**	**Renal Function**	eGFR ≥ 20–25 mL/min/1.73 m^2^	Safe to initiate empagliflozin or dapagliflozin (10 mg QD); monitor renal function regularly
**5**	**Volume and Nutritional Status**	Risk of dehydration (e.g., mucositis, vomiting, diarrhea), poor oral intake	Delay or withhold SGLT2is during active volume depletion or acute illness; reinitiate once stable
**6**	**Glycemic Status**	T2DM, risk for ketoacidosis	Monitor for euglycemic DKA, especially in T2DM or during fasting states; educate patient on warning symptoms
**7**	**Multidisciplinary Review**	Oncology, cardiology, nephrology input	Confirm indication and timing; integrate into broader cardio-oncology treatment plan
**8**	**Monitoring Plan**	Cardiac biomarkers, echocardiography, renal function	Monitor response and side effects every 4–6 weeks initially; adjust based on clinical status and cancer treatment course

* HFrEF: heart failure with reduced ejection fraction; ASCVD: atherosclerotic cardiovascular disease; GLS: global longitudinal strain; T2DM: type 2 diabetes mellitus; DKA: diabetic ketoacidosis; QD: once daily.

**Table 3:** Search strategy used in Medline and EMBASE.

Database	Search String
**Medline**	(“SGLT2 inhibitors” OR “gliflozin”) AND (“cancer” OR “cardio-oncology”) AND (“cardiotoxicity” OR “cardiovascular disease” OR “cardioprotection”)
**EMBASE**	(“SGLT2 inhibitors” OR “gliflozin”) AND (“cancer” OR “oncology”) AND (“cardiotoxicity” OR “cardiovascular disease” OR “heart failure”)

Note: Search terms were adapted to the indexing systems of each database. Boolean operators (AND, OR) were applied without truncation to ensure both sensitivity and relevance of results.

## Data Availability

Not applicable.

## Abbreviations

The following abbreviations are used in this manuscript:

SGLT2	Sodium-Glucose Cotransporter 2
SGLT2is	Sodium-Glucose Cotransporter 2 Inhibitors
T2DM	Type 2 Diabetes Mellitus
HF	Heart Failure
CTRCD	Cancer-Therapy-Related Cardiac Dysfunction
CTRCT	Cancer-Treatment-Related Cardiac Toxicity
BAT	Brown Adipose Tissue
WAT	White Adipose Tissue
MACE	Major Adverse Cardiovascular Events
CV	Cardiovascular
CVOTs	Cardiovascular Outcome Trials
NLRP3	NOD-, LRR-, and Pyrin Domain-Containing Protein 3
MyD-88	Myeloid Differentiation Primary Response 88
NF-kB	Nuclear Factor Kappa-Light-Chain-Enhancer of Activated B cells
IL	Interleukin
IL-1β	Interleukin-1 beta
IL-6	Interleukin-6
IL-8	Interleukin-8
IL-10	Interleukin-10
IL-17α	Interleukin-17 alpha
IFN-γ	Interferon-gamma
TNF-α	Tumor Necrosis Factor alpha
G-CSF	Granulocyte Colony-Stimulating Factor
GM-CSF	Granulocyte–Macrophage Colony-Stimulating Factor
iROS	Intracellular Reactive Oxygen Species
MDA	Malondialdehyde
4-HNA	4-Hydroxynonenal
pAMPK	Phosphorylated AMP-Activated Protein Kinase
ATP	Adenosine Triphosphate
ADP	Adenosine Diphosphate
Th1	T-helper 1 Cells
Th17	T-helper 17 Cells
Treg	Regulatory T Cells
P/O ratio	Phosphate/Oxygen Ratio (measure of cardiac efficiency)
HER2	Human Epidermal Growth Factor Receptor 2
ERK	Extracellular Signal-Regulated Kinase
JNK	Janus Kinase
MAPKs	Mitogen-Activated Protein Kinases
DOX	Doxorubicin
EMPA	Empagliflozin
EMPA-DOXO	Empagliflozin + Doxorubicin Combination
LS	Longitudinal Strain
RS	Radial Strain
EMPACT	Empagliflozin in the Prevention of Cardiotoxicity in Cancer Patients Undergoing Chemotherapy Based on Anthracyclines Trial
PROTECT	Potential Protective Role of SGLT2 Inhibitors for Chemotherapy-Induced Cardiotoxicity Trial
PROTECTAA	CardioPROTECTion With Dapagliflozin in Breast Cancer Patients Treated with AnthrAcycline Trial
DM	Diabetes Mellitus
HR	Hazard Ratio
CI	Confidence Interval
